# Foreign Summary

**Published:** 1839-07-20

**Authors:** 


### FOREIGN SUMMARY.

*Cæsarean Operation.*—The following report of the Cæsarean operation, mentioned by Dr. Harlan, in his last letter, is by Mr. Dubois, the operator:

Rosine Regnier, twenty-three years of age, three feet three inches in height, was admitted in the hospital on the 24th of January, 1839. None of the patient's family were below the middle size, and up to the age of nine years the child did not present anything abnormal in appearance or form. About this time she was attacked with eschars on various points of the body; different portions of the osseous system were removed, and it was only after the lapse of four years that the wounds were completely healed. From this time the child ceased to grow, with the exception of the head, whose volume appeared to be excessive. About ten months ago the parents of the girl made an engagement for her with a man who gained his livelihood by exhibiting "phenomena" to the vulgar; the girl soon became pregnant, in consequence of a speculation of her master, who thought that the accession of an infant dwarf would add mightily to the interest of his show.

On her admission into the hospital the date of conception was supposed to be about eight months back. Labour-pains set in on the 28th of March, and on the 29th the operation was performed, in the presence of a crowd of spectators. The first incisions having exposed, through the linea alba and peritoneum, the body of the uterus, a quantity of intestine, together with the bladder, immediately escaped through the wound. All attempts to return them into the abdominal cavity were fruitless, and as vomiting and hiccough, with slight faintness, set in, the bundle of intestines was pushed aside, and the anterior wall of the uterus divided. A considerable jet of blood immediately followed, from the insertion of the placenta, but the fœtus was soon extracted, and the uterus at once closed on itself. As the patient was extremely feeble, the intestines were now returned into the abdomen without being cleaned, and a few points of suture were applied. The general surface, which was at first cold, recovered its heat after the lapse of an hour; the vomiting was arrested by ice-cold drinks, and an opiate enema was thrown up.

The patient passed a quiet night, but the vomiting returned on the following day; the abdomen became tumid and painful; some leeches were applied; these did not produce even temporary relief; the vomiting was now constant; the face much altered; the pulse extremely feeble, and the patient sunk forty hours after the operation. On examining the body it was found that a commencement of union had taken place in the incision through the abdominal walls, to which a portion of the small intestine was loosely adherent. The remaining portion of the bowels was coloured from imbibition of a small quantity of effused blood, and the only traces of inflammation were some shreds of gelatinous effusion, which united the intestinal folds together. The antero-posterior diameter of the inlet of the pelvis was two inches five lines; the transverse four inches. At the outlet of the pelvis the measurements were—antero-posterior, 2.6; transverse, 3.2.—*Lancet, from Bul. de l'Acad. de Med.*, May 15.

---

*Antiquity of the Operation for Wry Neck.*—From the following passage from Ward's Diary, written between the years 1648 and '79, and lately published in the London Medical Gazette, it appears that the brilliant operation of Dr. Stromeyer was performed by a mountebank, in the seventeenth century:

"The mountebank that cutt wry necks, cutt three tendons in one child's neck, and hee did it thus: first by making a small orifice with his launcet, and lifting up the tendon for fear of the jugular vein—then by putting in his incision knife, and cutting them upwards; they give a great snapp when cut. The orifice of his wounds are small, and scarce any blood follows. Some are wry neckt from the womb; they only lay a melilot plaister to heal the wound; the plaister must bee a fresh one every day. As for the symptoms of this cutting, they are only these: that about a day or two after, the child will be sickish, some humour falling on the stomach of itt, as the mountebank says. When hee hath cutt itt, hee bends the child's neck the other way, and puts on a capp and a fillet tied to the capp, and so ties it under the armpitts, and so by constant bending the head that way, itt becomes straight and upe right."

---

*Clinical Lecture on Diseases of the Heart.* By Robert Carswell, M. D.—Gentlemen:—Although I have, on several occasions, directed your attention to the study of the diseases of the heart, and more especially to their physical signs, the importance of the subject induces me to offer you some additional observations on the cases which we have had an opportunity of observing during the last three months. You will, I am sure, be surprised to learn that nearly *one half* of the patients admitted during that period, with various diseases, have presented physical signs of the existence of anormal conditions of the heart. From the 1st of January up to this date (28th March) there have been admitted 30 female and 29 male patients, with various acute and chronic diseases. Of the former there have been 14 cases; of the latter 11 cases, in which physical signs of these anormal conditions of the heart were observed. From a tabular view of the physical signs observed in 25 recorded cases it appears that by far the most frequent consisted of morbid sounds, either single or double, principally heard at the base or apex, or in both regions of the heart at the same time, and accompanying the natural sounds of that organ. In seven or eight of these cases the morbid sounds were accompanied with a greater or less degree of increase of the impulse and sounds of the heart, heard over a greater or less extent of the chest, beyond the natural limits. In three cases only were the morbid sounds absent, the diseased conditions being manifested by the preternatural extent of the sounds and impulse alone of the heart. In one case no physical signs were observed during life, the disease of the heart (concentric hypertrophy) not having been detected till after death.

Of all the cases in which physical signs of the existence of anormal conditions of the heart were observed, six only were entered in the case-book as *morbus cordis*, the heart being the organ essentially and primarily affected, and its morbid states the cause of the complications, such as general congestion, anasarca, ascites, hydrothorax, bronchitis, and emphysema, which had compelled the patients to seek for relief.

Of the 19 remaining cases three were cases of rheumatism, three of phthisis, and the rest of various diseases.

We shall divide all the cases into two groups; the first group comprehending those entered as diseases of the heart; the second including those entered under their respective names; and endeavour to estimate the diagnostic value of the signs appertaining to each.

In the first group the physical signs referrible to the morbid conditions of the heart, were of such a character as to entitle them to be considered *pathognomonic* of organic disease of that organ. In the cases of Gibson and Shaw the *post-mortem*

appearances confirmed, in every particular, the accuracy of our diagnosis. In Gibson's case there were marked physical signs of hypertrophy, and a loud morbid sound heard to the left of the apex of the heart; and after death the size of the heart was found greatly increased, and the mitral valve in a state of permanent patentcy. In Shaw's case the impulse of the heart was too strong, and heard too extensively. A slight morbid sound was heard at the base of the heart along with the diastole; and to the left of the apex along with the systole. After death there were found hypertrophy of the walls of the heart, rigidity, shortening, and obliquity of the mitral valve, and perforation of one of the semilunar valves,—conditions which sufficiently explained the production, by regurgitation, of the morbid sounds, and the too strong and extended impulse of the heart observed during life.

In the case of Shiraffs, which I have already detailed to you on a former occasion, we have physical signs equally conclusive of the existence of organic disease of the heart and its valves.

In the case of William Tobin, which I have not yet brought under your notice, the physical signs of organic disease of the heart possess the same positive value as those of the preceding case. He has been in the hospital for upwards of three weeks. The impulse of the heart is too strong and too extended; there is extensive dulness over the cardiac region; and a slight morbid sound, heard at the base, accompanies the second sound of the heart. There were, besides, some congestion of the face, lips and tongue; œdema of the feet and ankles towards evening; palpitations, dyspnœa, orthopnœa, and slight bronchitis.

In the fifth case, viz. that of Halliday, the physical signs were those of hypertrophy and dilatation, in which the latter probably predominated. The chief symptoms were referrible to congestion of the lungs, in some degree of the brain, of the digestive organs, and of the extremities. In his case the organic affection of the heart wss not considerable, and probably remediable, but it was the obvious cause of the symptoms enumerated; whilst in those of Shiraffs and Tobin* (who are still under treatment, and greatly improved,) it is extensive, and of such a nature as to prove ultimately fatal.

In the sixth and last of the cases entered as disease of the heart, viz. that of Foreman, we had also unequivocal physical signs of organic disease of the heart. Of the history of this patient's case, I shall merely state, that about a year and a half before his admission into the hospital, which was on the 15th of January, he became subject to a hacking cough, and soon after experienced severe palpitation of the heart on any exertion, as in walking quick, or going up stairs, &c. These symptoms continued up to that time, and became gradually more and more severe. When examined, the impulse of the heart was found too strong and felt over too large a surface. The rhythm was irregular, with occasional intermittence. The first sound of the heart was replaced by a loud, rough and prolonged morbid sound, most distinctly heard near the apex. The second sound was heard somewhat morbid at the base of the heart. There was also a very strong purring tremour felt over the cardiac region, especially when the patient stooped forward. The dulness on percussion was not too much extended. From these physical signs it was clear that we had organic disease of the heart of at least a year and a half's duration. We had, in fact, a case of hypertrophy and dilatation of the walls of the heart, and a morbid condition of the mitral valve, and of the semilunar valves of the aorta. This patient left the hospital greatly better at the end of two weeks. The increased action of the heart became gradually less, and the anormal sounds diminished in intensity. He was bled to eight ounces from the arm on his admission, and took the solution of the iodide of potassium and tincture of digitalis, during the period stated.

Of the second group, comprehending 19 cases of disease, in which physical signs existed of anormal conditions of the heart, three of them were cases of rheumatism, three of phthisis, two of erysipelas, two of eczema impetiginodes, one of acne, of peritonitis, &c. of cephalalgia, of dysentery, of poisoning by opium, of abdominal congestion, of dyspepsia, of colica pictonum, and of lichen.

In some of these cases the diagnostic value of the physical signs can be ascertained with equal certainty and facility. In the three first cases, or those of rheumatism, we have the physical signs either of valvular disease alone, or these combined with those of hypertrophy. You will, perhaps, recollect the case of Mary Bye, admitted the 31st of January, with acute rheumatism, the history and treatment of whose case I brought before you formerly, and which was rendered peculiarly interesting from the fact of the anormal sounds of the heart having, after the space of a few days, considerably diminished, under the influence of the treatment employed. She had been twice bled from the arm, and had taken the acetous extract of colchicum three times a day, and a pill of calomel, and compound powder of ipecacuanha morning and evening, until the mouth had become slightly affected. It was at this time that the anormal sounds in the region of the heart diminished in intensity. They consisted of a double bellows-sound, heard with the first and second sounds of the heart.

As I have already given you the history and treatment of this case up to the seventh day, and as it furnishes a most satisfactory example of the cure of endocarditis, or of inflammation of the valves of the heart, I shall now relate its further progress and termination. On the 9th of February, the rheumatic inflammation of the hand, elbow and shoulder had lessened, but it had become worse in the ankles, which were very red, hot and painful. The patient could get no sleep at night. The morbid sounds of the heart were the same, that is, less marked than when first

*Both patients left the hospital relieved of all the complications, and in their ordinary state of health.

heard, but still readily distinguished; and as the pulse was full and firm, twelve ounces of blood were again taken from the arm. The acetous extract of colchicum was increased to two grains, three times a day, and twenty-five minims of the tincture of the meconate of morphia ordered to be taken at night. On the three following days leeches were several times applied to the joints with considerable benefit. The colchicum had been increased to two grains and a half, and was followed by frequent vomiting and purging, and was, therefore omitted. The mouth continued sore; the inflammation and pain of the joints gradually diminished, by subsequent applications of leeches, and on the 23d were quite gone. On the 26th the morbid sounds of the heart had almost entirely disappeared, and six days after no trace of them could be heard, when the patient was discharged quite well.

That the double bellows-sound in this case indicated the presence of valvular disease, and that the morbid condition in which it originated was rheumatic inflammation, can no longer be matter of doubt to the stethoscopist; and much, if not the greater part, of the accuracy of our diagnosis of the presence of this complication of rheumatism, we certainly owe to the researches of M. Bouillaud, on what he has appropriately denominated endocarditis, in contradistinction to inflammation of the pericardium, or pericarditis. After having laid before you the two other cases of rheumatism, accompanied with physical signs of disease of the heart, I shall, if our time will permit, make some remarks on the seat of the morbid sounds heard in each, and on their probable cause and mode of production.

The second case of rheumatism, accompanied with anormal sounds, occurred in a boy, eight years of age, who was admitted on the 19th of February. Previously in the enjoyment of good health, he was exposed to cold, and was seized with pain in the ankle and foot of the right side, which afterwards shifted to the knees, and from them to the shoulders, arms and hands. When admitted the pain was confined to the left shoulder, unaccompanied by redness or swelling; was increased by cold, relieved by heat, and worse towards evening. His sleep was disturbed; skin hot; pulse 90; bowels irregular. On applying the stethoscope a bellows-sound was heard with the first sound of the heart at the apex only, and when respiration was suspended. The second sound was slightly morbid at the base; the rhythm was regular.

This case of rheumatism was altogether mild; neither the local nor general symptoms had been severe, and were, indeed, very slight when the patient entered the hospital. Notwithstanding, it was complicated with endocarditis, as shown by the stethoscopic signs which I have mentioned. Whether these disappeared before the patient left the hospital was not ascertained; he was discharged, cured, a week after admission, the chief means employed being the solution of the iodide of potassium.

The last of the cases of rheumatism, complicated with disease of the heart, is that of James Finnagen, ætat. 33, admitted 19th of February, and is still under treatment. He is a tailor by trade, married, of pretty regular habits, has been lately much exposed to cold and wet, and much fatigue. He has always had good health (with the exception of a cough during the winter months of the last five years,) until eighteen months ago, when he became subject to a continued pain in the cardiac region, aggravated at times, and great palpitation. This cough became worse, and the expectoration more profuse. He had supra-orbital headach, red clouds and flashings of light before his eyes, defective vision, and sometimes dyplopia; giddiness, and sometimes he fell insensible; tinnitus aurium, and frightful dreams. These symptoms continued until three or four weeks ago, when he got wet through for several days together, and soon experienced pain in his knees, ankles, hips, loins, left shoulder, elbows and wrists. The pain also in the cardiac region became worse. He had no advice for the rheumatism, but applied at Moorfields on account of the dimness of sight. He was given some ointment of tartarised antimony to rub on the back of his neck, and had some blue pills to take, but his mouth becoming sore, he discontinued the use of both. He got gradually worse, and on the 19th of February came to this hospital.

*Present symptoms.*—Skin hot and dry; tongue white; headach; disturbance of vision, and dreaming as before-mentioned, with pain in the cardiac region, increased on firm pressure between the ribs; palpitation, with cough and slight expectoration; pulse 84, full and strong; bowels regular; urine scanty and high coloured.

A double-bellows sound (the second the louder) heard most intense at the junction of the fourth left costal cartilage with the sternum, and also over the whole cardiac region; impulse much too extended; rhythm regular; sonorous rattle in both lungs anteriorly, but most marked in the left.

The principal signs and symptoms observed in this case were obviously those of rheumatism and endocarditis (those indicating cerebral disturbance and bronchitis we shall pass over for the present.) The acute affection of the joints and endocarditis existed together at the time the patient was admitted, the former having occurred about three or four weeks previously, after exposure for several days to wet and cold. The latter, or the endocarditis, most probably existed from the period stated in the report, viz. eighteen months prior to the attack of rheumatism, when the symptoms then felt by the patient were aggravated. Great palpitation and continued pain in the region of the heart were the principal symptoms which accompanied the cardiac affection at that time, and which continued more or less up to the time of the rheumatic attack of the joints. In addition to these symptoms, and the double bellows-sound heard at the base of the heart, there were, also, when the patient was admitted, marked signs of hypertrophy of the left ventricle. Whether this complication was the consequence of the endocarditis which occurred eighteen months ago, or existed previous to that period, we cannot now ascertain with certainty. But as it is stated in the history of the case that the patient had always enjoyed good health up to that time, it is extremely probable that the acute affection of the heart, or endocarditis, which then manifested itself by the symptoms which I have stated, preceded the hypertrophy which we found to exist when the patient was submitted to our examination. There is no doubt that hypertrophy in this, as in many cases, may have existed before, and favoured the development of the endocarditis. However, as the latter disease is by far the most frequent of the causes of hypertrophy of the heart in the young, and those of middle age, beside its probable occurrence in this case in a subject susceptible of rheumatic inflammation from exposure to cold, I am disposed to believe that the hypertrophy occurred subsequently to, and as a consequence of, the endocarditis.

This is by far the most severe of the three cases of rheumatism with complication of cardiac disease. The patient, at the period of this report, was under treatment six weeks, and although the symptoms of the rheumatic and bronchial affections soon disappeared, and the cerebral and other symptoms greatly diminished in severity, the anormal sounds and hypertrophy of the heart were as strongly marked as ever. The action of the heart was still equally strong and extended, and the bellows-sound which occurred during the diastole was so strong as to obscure entirely or replace the second sound of the heart. Under these circumstances, we cannot entertain the slightest doubt that there exists in this case permanent organic disease of the valves and walls of the heart. The treatment consisted, at first, of general bleeding, cupping and leeching, blistering and colchicum; and afterwards of the iodide of potassium.

Of the sixteen remaining cases in which physical signs of anormal conditions of the heart were observed, in at least two of them were these signs indicative of organic disease. In the first of these, the disease for which the patient, a female, was admitted, was acne. Besides the physical signs, which were those of hypertrophy and dilatation, as shown by the increased impulse of the heart and the extent of the chest over which the anormal sounds of that organ were heard, there were symptoms of general congestion, but more especially of the brain. In the second case, which was one of colica pictonum, there was not only increased impulse, but a bellows-sound heard with the first sound of the apex; intermittence and irregularity of the heart's action.

In the other two cases already alluded to,—one of phthisis, the other of peritonitis,—the nature and extent of the organic affection of the heart were observed by us after death.

Twelve cases now remain of the whole number, viz. twenty-five, the physical signs observed in which were the following:—A slight bellows, or slight morbid sound, heard at the base or apex of the heart, or at both, constituted the only signs that were observed; in one case the morbid sound was rough and scraping at the apex, and in another it was double, heard equally at the base and apex, and at the top of sternum.

In none of these twelve cases did the morbid sounds appear to indicate extensive disease. Not only were the physical signs which they furnished mostly slight in degree and extent, but they were unaccompanied by any symptoms or complications which could be considered to have any connection with them as signs of cardiac disease. That they were, nevertheless, signs of the existence of some morbid condition of the heart is more than likely, from the most of them having been heard on several occasions during the stay of the patients in the hospital, consequently under different conditions or circumstances; from their occurring in male as well as in female patients (in three of the former and nine of the latter;) in diseases of a very different, and even opposite kind; in patients from two up to fifty-eight years of age, and in none of whom were present those nervous, and more especially anemic states which give rise to the production of anormal sounds in the heart.

In two of the patients, fifty and fifty-eight years of age respectively, the anormal sounds might, from the absence of all other predisposing or exciting causes, in the history of either case, be reasonably supposed to depend on some one or more of those morbid alterations which make their appearance so frequently in the valves of the left side of the heart, about this period of life, and as a consequence of age. I must, however, observe that perhaps too mı ch has been attributed to this circumstance alone, in our desire to explain and account for the frequent occurrence of fibrous, cartilaginous, and osseous transformations in the heart and arterial system, after a certain period of life. It is, certainly, a well-established pathological fact, that such alterations do occur in accordance with the law of pathological transformation of analogous tissues; and nowhere is it so frequently verified as in the heart and arteries, without our being able to trace it to such a cause as inflammation. Still, as by far the greater number of analogous tissues and transformations can be demonstrated to originate in inflammation, we shall not err if, in the absence of more direct evidence, we regard these lesions of the heart, and of the valves especially, which give rise to the production of anormal sounds, as having a similar origin, not only in all cases at an early period of life, but very frequently when the progress of age is supposed sufficient to explain their occurrence.

The remarks which I have already made on the physical signs observed in these twenty-five cases of cardiac disease, and the remote origin of by far the greater number of them in inflammation, render it unnecessary for me to enter into a minute investigation in regard either to the precise part of the heart affected, or the nature of the change which it had undergone.

Although we are, perhaps, not yet in possession of the means of ascertaining with certainty the precise locality of the morbid sounds developed in the region of the heart, yet I am disposed to believe that we can do so in the great majority of cases, and certainly with sufficient accuracy for all practical purposes. The *character* of the morbid sounds, the *situation*, and *direction* in which they were best heard, were such in the most of our cases as to leave little doubt of their depending on *valvular* disease. The morbid sounds were chiefly of the bellows kind, and, as I have already said, were heard in the situation of the semilunar valves, in the direction of the aorta or upper part of the sternum, and in the situation of the mitral valve, towards the left, or at the apex of the heart. I do not believe that we have had unequivocal signs of pericarditis having existed in a single case, out of the twenty-five recorded cases,—an approximative fact at least, which is little in accordance with the opinion formerly, and not long since entertained, of the great frequency of pericarditis as a complication of rheumatism, or as an idiopathic disease. A still greater frequency, however, has been ascribed, especially by M. Bouillaud, to the occurrence of endocarditis in rheumatism, this author having asserted that inflammation of the lining membrane of the heart always accompanies rheumatic inflammation of the joints. This is certainly an exaggerated statement, in so far, at least, as it rests for support on the presence of physical signs. For, among the rheumatic cases admitted into our wards during the last three months, we have had four cases of acute rheumatism in which no physical signs of endocarditis, or other affection of the heart, were observable; and also two severe cases of sciatica under similar circumstances. From the facts, however, which I have stated, it is but too certain that endocarditis is an extremely frequent complication of acute rheumatism; and in no case of this disease, however slight the local affection and the general symptoms, should we neglect to examine most carefully the condition of the heart, during the whole course of the disease.

It is by no means rare to meet with mild cases of acute rheumatism, for which active antiphlogistic treatment is considered not at all necessary, and yet in which there may be signs of endocarditis requiring the employment not only of this means but of the most powerful agents we possess of arresting the progress of this serious complication, and of which mercury is the most certain and efficacious.

I shall not insist farther upon the practical importance of a knowledge of the physical signs of endocarditis, without which, you must perceive, you never can ascertain the existence of this complication in rheumatism, nor, consequently, secure your patient against its consequences at some future period of his life.

I should now say a few words on the nature of the morbid states of the valves, particularly those consequent upon inflammation; and on the production of the morbid sounds to which they give rise, and by means of which their existence is detected; the consideration of both of these subjects, however, I shall defer to some more favourable opportunity.—*Lancet.*

---

*Remarks on the removal of Tumours by Deligation.*—At a late meeting of the Royal Medico-Chirurgical Society of London, a paper was read by Mr. Liston, on this subject, the object of which was to afford an additional recommendation of a practice, applicable to the ligature of vascular tumours, which has been again and again laid before the profession. The author is induced to bring it forward on the present occasion, because it was not noticed in either of two papers on nævus read lately before the Society. The plan alluded to consists in dividing the skin at the base of such tumours before tightening the ligature, in order to avoid including it in its noose. By thus modifying the operation, the author considers that several objections to the use of the ligature are avoided; namely, the intense suffering which attends its application in the ordinary way; the imperfect strangulation of the included mass; the bleeding which sometimes occurs on the separation of the sloughs; and the deformity caused by the destruction of the cutaneous and other tissues.

Dr. Marshall Hall observed that he was still of the opinion that he had formerly expressed, viz., that the treatment by means of a very small knife, or cataract-needle, was that to be preferred. It was unattended by pain or hæmorrhage, and was not followed by scar. If repeated often enough, and if time enough were given for nature to work her changes, it would effect everything that we could wish. We had, in fact, only to replace the nævous structure by cicatrix, and the cure must be accomplished. Now, this might be done without sacrificing the skin, or inflicting more pain than the prick of a needle, and without inducing hæmorrhage. Everything might be done under the skin, and preserving the skin, which could be effected by ligature, excision, or caustic. We might pierce the nævus from one point, in various directions, in the first instance; break it down fearlessly, in the second; and we might, by a little care, even apply a ligature, preserving the skin; for this purpose, a proper instrument, armed by a ligature, might be made to pierce the nævus near its edge, or to pass along one-half of its circumference, then to pass out at the opposite edge, and be introduced as before, and passed along the other half of the circumference; it might then be tied, and it would simply involve the nævus structure, exclusively of the skin. He repeated that everything that could be done sacrificing the skin, may be done preserving that structure, and, by doing so, avoiding pain which has induced convulsions, hæmorrhage which has induced fatal exhaustion, and a frightful scar. The only objection to this mode of operation was the time required, and the impatience on the part of parents and practitioners the only source of failure. He believed the profession were still in ignorance of all that it was calculated to achieve.

Sir B. Brodie said that he was surprised that in neither of the papers on nævus read this session, nor in the discussions upon them, had any allusion been made to the very simple mode of treatment by pressure. A great number of subcutaneous nævi could by this means be effectually cured. In the case of a very large subcutaneous nævus on the back, in which he was divided in opinion whether to use the knife or ligature, he determined, eventually, to try the influence of pressure. A piece of ivory, rather larger than the nævus, was placed over it, and there secured by sticking-plaster and a bandage; the pressure exerted was not great; the nævus entirely disappeared under this treatment. In another instance, in which the nævus was situated on the crown of the head of a child, very near to the fontanelle, no operation would have been advisable; the head was shaved, a piece of sheet-lead applied over the nævus, and kept on by strips of adhesive plaster; in a few months the tumour disappeared. This plan was applicable to many cases of subcutaneous nævi. Occasionally, also, it might be serviceable where the skin was slightly involved, but in cases of pure cutaneous nævi, sloughing would follow the application of pressure.—*Ibid.*

---

*Clinical Lecture on Osteitis of the Upper Third of the Femur, simulating Hip-joint Disease.* Delivered in St. Vincent's Hospital, Dublin, by J. M. Ferrall, Esq., M. R. I. A., First Surgeon to the Hospital.—"Disease of the hip-joint," says Mr. Ford, "in every stage of its progress, from its earliest appearance to its final catastrophe, is marked throughout by peculiar and characteristic symptoms." Such, gentlemen, is the opinion of the author of the most original and not least accurate history of hip disease that has, perhaps, ever appeared. I have, however, had occasion, more than once, since the commencement of the present session, to call your attention, in the wards of this hospital, to a disease of a nature altogether different from morbus coxæ, and yet presenting so many and such striking points of similarity to the latter affection, that the tact and experience of able men have, as you yourselves have witnessed, been foiled by the resemblance.

There is a case of this kind at present under our view; and as there are also two cases of morbus coxæ under treatment in St. Patrick's and St. Joseph's wards, I have selected this subject for the matter of our present inquiry.

The disease in question, as far as I have had opportunities of observing it, is met with in an acute and a chronic form. The case at present in the hospital is of the former character; it will be more convenient, therefore, to commence with the history of that variety of the complaint to which it belongs.

In the acute form the disease commences rather suddenly, and without any previous indisposition observable by the patient. He may retire to rest after the enjoyment of exercise, without even the feeling of fatigue, and be disturbed during the night, or awaken in the morning, with severe pain, referred to the hip, and extending to the groin and knee. The pain is so much aggravated by any attempt at movement, that he is rendered perfectly lame; and in severe cases the effort to use the limb may be so very painful that he is totally unable to leave his bed. In the humbler walks of life, however, where little alarm is excited by the mere occurrence of pain, the child may be suffered to struggle to the erect or sitting posture every day for one or two weeks, until his altered appearance becomes too striking to be overlooked. During this period a state of fever is continually present; the pulse is very frequent; the skin hot; thirst urgent, and tongue coated; the urine is scanty and high coloured; there is flushing, occasional headach, and tendency to vomit; the severity of the pain may be less constant, or may undergo periodical exacerbations, generally occurring towards evening, or in the night. The sleep is disturbed, and accompanied by moaning and evident distress; or, in aggravated cases, interrupted by delirium. The symptomatic fever is, in fact, generally well marked; and it is worth your remembering that it may run so high as altogether to mask the local disease. I saw a case in Dorset street, in September last, where delirium was the prominent symptom, and this lesion of the intellect deprived the medical attendant of all information as to the suffering endured by the patient for several days, during which time the real nature of the complaint was not even suspected. Such are the early symptoms of the complaint, which in very acute cases are manifest to the patient or his friends: when the fever is less urgent, and a state of feverishness, loss of appetite and rest, are the general symptoms, the little patient is generally allowed to creep about, although unable to use the limb. If you are consulted at this period of the disorder, two or three weeks from the accession of the attack, you will perhaps be told that perspirations are beginning to be added to the fever, and that he has lost flesh considerably. On inquiry, the pain is referred to the hip about the trochanter to the groin and knee, sometimes as if passing through the joint, and occasionally extending lower down on the outside of the leg a few inches below the head of the fibula, and accurately defined. If you now proceed to examine the limb, the following will be found to be the phenomena:—

If the patient be in bed, he will generally be found lying with the thigh fixed on the pelvis, and inclined towards the mesial line. The comparative length of the two limbs is a point not easily ascertained, on account of the bent position of the one affected; but one knee has sometimes appeared to project more than the other. The loss of power in the muscles about the hip is evident when he alters the position of the limb, for you will observe he accomplishes this object by insinuating the opposite foot under the ankle of the diseased side, and lifting it to the place intended for it. Pressure, even lightly made, on the trochanter or groin, causes pain, and this pain occasionally extends as far as the knee.

In the erect posture there is complete inability to sustain the weight of the body on the affected limb; the knee is advanced, the toe resting on the ground, and the heel supported against the front of the other leg. The pelvis is obviously lower on the affected side; the nates of that side is flattened, and sinks; the obliquity of its lower margin presenting a marked contrast to the horizontal level of the sound one; the muscles of the affected leg are wasted in a remarkable degree. If, while standing before you, he changes his position to either side, or is lifted to the right or left, the sound leg is at once placed in the line of the centre of gravity, to support him; but the affected limb either remains as it was before, or is carried after its fellow by the patient, who instinctively raises it by one or both hands, and places it in its new position.

So far, gentlemen, the phenomena resemble those of acute disease of the hip-joint so closely, that you might easily suppose I had been describing the latter disease; but a more searching inspection will develope other phenomena capable of correcting the first impression, and suggesting a more precise view of the nature of the complaint.

In the recumbent position, for instance, the limb is generally found flexed upon the pelvis. Now, this is not the position usually adopted by patients in the early stage of morbus coxæ; and at the period when it does generally occur, the articulating surfaces are engaged to an extent to render the least motion of the head of the femur in the acetabulum a source of severe pain. In the disease we are now considering, if you take the limb so gently in your hand as not to call into action the muscles which connect it with the trunk, and then press it firmly into the acetabulum, no complaint is made. You can, with the same precautions, cause it to perform all the motions of flexion and extension, adduction and abduction, without the slightest uneasiness to the patient. These are movements which, in true morbus coxæ, would be agonizing in that stage when the limb is found to be habitually in the flexed position.

You will, no doubt, meet with cases of the scrofulous disease of the hip-joint, in which these passive movements can, in the early stages, be performed without any remarkable expression of pain; and, therefore, this test, taken singly, loses much of its value. Taken, however, in combination with that which I am next to mention, it will assist materially in the inquiry. If, while you cause the limb to perform those passive motions of adduction, abduction, flexion, and extension, you fix your eye upon the anterior superior spinous process of the ilium, you will perceive that this bony prominence remains at rest while the femur moves, and that the head of the latter plays freely in the acetabulum. Now observe what takes place in the genuine hip case, when this manipulation is performed by the surgeon. Every movement of the femur is accompanied by a corresponding change of position of the anterior spinous process of the ilium; the pelvis moves with the femur; and both together now constitute an angular lever, the fulcrum of which is at the acetabulum of the sound side, and the motions of which are performed on the head of the sound thigh-bone. This symptom of inflamed hip-joint has not, I believe, been before described; and I have looked carefully into authors with this view since I first observed it. I have examined a vast number of cases of hip disease, and have not yet met with an exception to the rule of its occurrence. This movement of the pelvis with every motion of the femur, in morbus coxæ, will explain certain cases where little or no pain is complained of by the patient, the motion in many of those instances being only apparent as regards the joint, and the deception being occasioned in the manner I describe.

You can render this fixity of the hip in morbus coxæ more manifest by the following manœuvre:—Place the patient on his back; take one of his ankles in each of your hands, and, while you separate the limbs as far as they can go, observe accurately the two fixed points of the pelvis—the spinous processes of the ilium. You will observe the femur of the healthy side plays freely in its socket; and further, the limb is abducted without any change in the direction of the foot. On the other hand, the diseased limb carries with it the pelvis; and the latter not only follows it, but is rotated, together with the spine, towards the opposite thigh, in such a manner that the foot is no longer directed forwards, but is inclined towards the middle line of the body. It is difficult to describe this contortion; but as you have witnessed it in both the hip cases now in the hospital, you require no further aid to understand it. In hip-joint disease, in fine, motion of every kind, active and passive, is a source of pain; while in osteitis of the upper end of the femur, although the patient is in the early stage incapable of moving the limb, no pain is experienced when this motion is accomplished by another.

The *rationale* of these opposite conditions is, I apprehend, to be sought for in the muscles of the parts. In the one case a tonic contraction of the muscles connecting the pelvis and femur is set up, as it were, to maintain the repose of the joint, and prevent the irritation inseparable from its disturbance. In the other case every movement which is painful is caused by the traction of the inflamed periosteum and tendinous attachments adjoining it, when the muscles are called into action. A state of the muscles therefore exists totally different from the former, and is more allied to their powerless condition in acute muscular rheumatism. Passive motion, is in such cases, freely permitted, for the same reason which prevents every active effort being attempted, namely, the muscular action is a source of pain.

If you now seek for diagnostic marks in the erect posture, you will observe that the limb, which is emaciated at the nates, calf of the leg, and lower part of the thigh, presents at its upper third a remarkable expansion; a sudden enlargement occurs here, which gives this portion of the limb a conical figure, the base being placed towards the hip-joint. This swelling is firm to the touch, and is evidently deep-seated; it is, in fact, enlargement and thickening of the bone and its coverings.

The observations already made with respect to the movement of the thigh-bone independent of the pelvis, in the recumbent position, are equally applicable to the erect posture. In the hip case, on the contrary, if you try to extend the thigh on the pelvis, while the patient stands erect, you will observe the pelvis moving backwards every time, and the lumbar spine more curved at the same instant, to accommodate itself to the other parts.

You will by these means have now acquired positive and negative evidence on which to rest your opinion of the nature of the case. You will have, on the one hand, the existence of a visible and palpable swelling of the upper third of the femur, which marks the existence or actual disease of that bone; and, on the other, you will have ascertained that passive motion can be performed without pain, and independent of the pelvis, to a degree inconsistent with the history of hip-joint disease. Much patience will, however, be required in examining the parts before an opinion is pronounced. Sir B. Brodie, whose tact in the discovery of obscure disease is acknowledged, and whose experience in this class of diseases is perhaps unequalled, expresses himself forcibly on the insidiousness of complaints about the hip-joint. In the present inquiry it is obvious that the evidence by which we are enabled to distinguish the disease of a bone in the vicinity of other joints is in a great measure absent here. If the the lower end of the femur be enlarged, and pain in the knee be complained of, we are led to the real nature of the complaint by the obvious disproportion between the femur and tibia; but when the upper end of the bone is engaged, all comparison is prevented—in the first place, by its being articulated with a flat bone; and secondly, by the depth at which it lies, as well as the thickness and increasing size of its coverings at this part.

The first stage of the disease, to which the foregoing observations apply will generally last some weeks, differing in this respect according to the acuteness of the attack.

The second stage is marked by the occurrence of a more irregular fever, preceded by slight rigors, and ending, though not constantly, in perspiration. The countenance acquires an anxious and haggard expression, such as you observe to occur in hectic fever, and the hair has the moist and stringy appearance of that state. The skin is generally hot, and sometimes dry, sometimes perspiring; the pulse ranging from 112 to 120; the urine occasionally depositing the lithates and purpurates. The tongue may be coated, yet florid at its edge; but I have too often impressed upon you, that the states of this organ will necessarily depend on the condition of internal parts, especially of the digestive tube, to render it necessary to repeat, on this occasion, that, taken as a symptom, no connexion can be established between its various appearances and the existence of any so-called surgical disease.

The local phenomena will, at this period, have undergone some modification. The swelling has increased, and some portion of the integuments have become more prominent, or have acquired a blush of inflammation. Here you may generally expect the formation of matter, although there is neither swelling nor fluctuation in a degree analogous to what occurs in suppuration depending on the soft parts alone. The patient remarks, that just when on the point of falling asleep, he is disturbed by twitching and starting of the affected limb, and his rest is sometimes altogether broken in this manner.

At length the matter is evacuated, either spontaneously or by the assistance of the surgeon, and a remarkable calm ensues. The pain in the hip, groin, and knee, is mitigated; the rest is restored; the fever abates, and the perspirations cease. The appetite revives under this favourable respite from suffering, and the countenance begins to improve, although the pulse may retain its frequency. The inflammation having by this time in a great measure exhausted itself in the organic changes effected in the bone, as well as in the suppuration in the adjacent parts, the morbid sensibility of the attachments of the muscles abates, and the patient begins gradually to be capable of moving the limb to a certain extent without pain. Although the comparative freedom from pain, and capability of some degree of active motion, characterize this second stage of the complaint, the position of the limb, when at rest, is nearly the same as at first. It is flexed on the pelvis, and the latter is found to be still awry, the ribs and ilium being closer on this side than on the other. The muscles appear to be more at ease in this posture; but, unlike what occurs in the inflammatory stage, they are now capable of moving the limb to a nearly straight position by their own unassisted powers. During the progress to the third stage, occasional accessions of fever and pain are to be expected. The pain is to be referred to the same points—namely, the hip, groin, front of the knee, or directly through the joint. I have heard the same person complain at one time of the outside of the knee and upper part of the leg, and at another refer it to the situation of the inner condyle. These exacerbations are sometimes the forerunners of fresh formations of matter; and, when this is the case, are generally relieved by its exit. They may, however, pass off without this result, especially if a few leeches have been applied. When new abscesses occur, the place of pointing is most frequently on the outside of the limb, in the neighbourhood of the great trochanter; although I have frequently seen them at the back of the thigh, below the nates. The fistulæ are, for the most part, so indirect and circuitous, that you cannot readily trace them to their termination. The bulk of the swelling is at this time found to be increased; the trochanter being also enlarged in all directions, will appear to ascend higher on the ilium than in the natural state. The remainder of the limb being more emaciated, increases the appearance of disproportion between the upper and middle portions of the bone. The integuments of the whole limb are sometimes thickly covered by a long fine hair, such as is occasionally seen in diseases of a marasmal character. The sound limb may exhibit a similar growth, but in much slighter degree.

If you try the effects of passive motion at this period, you will find that although flexion of the thigh on the pelvis is as extensive as before, you cannot now extend the limb in an equal degree. The flexed position maintained by the patient, in order to relax the muscles inserted into parts engaged in the morbid action, has now become habitual; partly owing to a contracted or shortened state of those muscles, in consequence of their long-continued unnatural position, and partly to a mechanical obstacle to extension presented by the increased growth of the femur coming in contact with the brim of the acetabulum. Abduction is nearly as free as in the early stage, but adduction appears to meet also with a firm resistance. The sudden check experienced is easily interpreted by the surgeon, who remembers the hypertrophy of the bone at this part. Some pain is occasionally experienced at the moment when the solid obstacle to adduction is perceived, but it is slight, and referred by the patient to the part struck alone, and not to those distant parts which suffer by sympathy or nervous communication. I must observe, that I have seen a case in which the period occupied in other instances by these changes has passed over without any formation of matter or opening in the integuments.

The third stage of this affection may be said to comprise the period in which the curative process is in progress; the nature of the changes included in this effort of nature, depending, of course, on the degree of injury inflicted by the violence of the primary disease. If many portions of the bone have lost their vitality, their separation from the healthy parts is to be accomplished; and other organic alterations, of a kind not so easily repaired, must have undergone a favourable change before the patient is restored to health. It is in this stage that the powers of the constitution are put to the severest test, and that the disposition to tubercular disease is most likely to be developed.

During this stage the fistulous openings continue to discharge pus. The qualities of this fluid will be found to differ materially at different times, and even at the same time from different orifices. I have seen it remain ill-coloured and fœtid for a considerable time, from one or two of these openings, while a more healthy matter was secreted from the remainder. When this has occurred, without being followed by any exfoliation, and has, after a long interval, gradually changed into a more healthy discharge, I have been led to suspect that caries of that portion of the bone corresponding to this peculiar secretion had existed; but as we are not in possession of evidence by which we can determine the question of absorption of dead bone, I cannot assert that the unfavourable condition of the fistula may not have depended on the presence of a loose piece of bone which had gradually disappeared, and not on ulceration properly so called.

The alteration in the discharge alluded to, is sometimes accompanied by increase of swelling and tenderness about the orifice, as well as by some degree of symptomatic fever. It has occasionally happened that these symptoms have been succeeded by the discharge of a portion of sequestrum, generally small in size, and more porous than those which you see eliminated from necrosis in the middle of a long bone. You will, on some occasions observe this exfoliation to be announced, not by any alteration in the quality of the discharge, but by its total cessation for a few days; and I think I have remarked that the pain and local irritation is then more severe than in the former case.

You may meet with a case where, after the formation of an abscess, the opening will remain solitary for several months; and if the probe be used to test the condition of the bone, you may be foiled by the length and curve of the fistulous canal, and probably obtain no satisfactory information as to its actual state. The swelling may remain undiminished, the only change being that it is less tender on pressure, and that the patient has acquired more power over the limb. The constitutional symptoms, in such a case, may be as stationary as the local complaint. There may be no sweats, nor accessions of fever; but although the appetite and rest are nearly natural, there is not much progressive improvement in the appearance of the invalid. As there may not, during this time, have been any exfoliation, there is no certainty that such a case is one of necrosis; and as I have not yet had the means of ascertaining the anatomical characters of a case presenting exactly the symptoms I have described, I merely mention the facts, and reserve their explanation to another occasion, when an opportunity shall have occurred of tracing the history to its termination.

The cases which I have seen sink in this stage have not done so under the influence of this disease alone. They have died of pulmonary disease. Those which have evinced sufficient vital powers to sustain, and, it may be, to subdue, the force and tediousness of the complaint, are yet under observation. Both these classes include cases in which treatment has been either ineffectual or untried. Another class, in which the attack has yielded to early and active remedies, remains to be spoken of when we come to discuss the treatment of the disease.

This, gentlemen, is nearly the history of those cases which commence in the acute form, and bear, in their phenomena, the closest analogy to inflammation, with necrosis of the long bones, modified, of course, by its occurrence in a part more highly organized, and resisting, therefore, to a certain extent, the destructive power of severe inflammatory action, which, in more compact osseous tissues, occasions the complete and extensive death of the parts.

The cases to which I have hitherto drawn your attention, are, I believe, instances of genuine osteitis, by which I apprehend must be understood inflammation of the cellular tissue by which the bone is pervaded throughout. In those cases, not only the periosteum, but the cellular tissue connecting that membrane with the bone, and that which conveys the vascular nett-work to the interior, become the medium for conducting this formidable disease to the spongy tissue. There are, however, instances to be met with, in which the periosteal covering of the bone is alone affected; but as the latter form of the disease does not, as far as I have observed, suggest a different mode of treatment, I do not, on this occasion, deem it necessary to detain you by a separate consideration of the periostitis of the upper third of the femur.

I may, however, remark, in passing, that the swelling is never so great when the periosteum alone is engaged, although the symptoms which simulate so closely disease of the hip-joint, depending, as they do, on the condition of parts connected with the surface of the bone, will generally be present in an equal degree.—*London Medical Gazette.*

### ANTI-NEURALGIC MIXTURE

### Of Dr. Liegard, of Caen.

℞. Aquæ Lactucæ,
Aquæ Lauro-cerasi, āā ℥ij.*
Extr. Lactucæ,† gr. xv.
Extr. Belladonnæ, gr. vj.
Extr. Hyoscyami, gr. viij.
Extr. Stramonii, gr. x.

### POTION, BY THE SAME.

℞. Aquæ Lactucæ, ℥j.
Aquæ Lauro-cerasi, ℥ij.
Extr. Lactucæ, gr. xij.
Extr. Belladonnæ, gr. vj.
Extr. Stramonii, gr. viij.
Extr. Hyoscyami, gr. x.
Syr. Valerianæ, ℥j.

The potion is to be taken in the dose of a teaspoonful once, twice, or thrice a day; the dose of the mixture is from five to six drops in sugar-and-water, when taken internally, and a teaspoonful when used externally. These two methods of administration are employed at the same time.—*Ibid, from Gazette des Hôpitaux.*

* As the French *gros* contains seventy-two grains, it would be more exact to translate this quantity by gr. cxliv.

† In the original it is Extrait *de Thridace;* but as *thridace* is itself an extract of lettuce, this is probably an error.

*Large Doses of Active Remedies.*—M. Forget, professor in the medical school at Strasburgh, has lately published, in the *Bulletin de Thérapeutique*, several interesting cases, showing the *tolerance* of some remedies in extraordinary doses.

Case I. *Tartar emetic.*—A robust butcher, aged forty, labouring under acute articular rheumatism, took tartar emetic in a potion, first in the dose of eight grains, then ten, fifteen, twenty, thirty, forty, sixty, and seventy-two grains, (a drachm,) and this without any disorder of the intestinal canal, or any other bad symptom. The tongue was moist, but whitish; the patient always ate quarter diet, but became disgusted with the potion, and it was discontinued. Thus in the space of ten days, he took, without inconvenience, about three drachms of tartar emetic. Nevertheless he had a relapse of the rheumatism, but was cured by colchicum wine.

Case II. *Pomegranate root bark.*—A girl of four-and-twenty, fair, and of a lymphatic temperament, had suffered from tænia since her childhood, and frequently passed fragments of the worm in her stools. She took two ounces of the bruised bark of the pomegranate root boiled in two pounds of water, at thrice, with half an hour's interval between the doses, but without effect. The dose was now increased to three ounces, and two tapeworms were expelled; so that in two days, and without any abdominal disturbance, the patient took the decoction of five ounces of the bark of pomegranate root.

Case III. *Ioduret of starch.*—Dr. Buchanan, of Glasgow, in 1836, published in the London Medical Gazette, some remarks on the exhibition of this substance in the enormous dose of seventy-two grains and more, without any bad symptom.

M. Forget tried this remedy on a youth of seventeen, labouring under lymphatic turgescence, with ganglionic tumours of the parotids, scrofulous ulcers, &c. The iodine was first given in the dose of twenty-four grains, with an ounce of starch. The iodine being triturated with a glass of water, was carefully mixed with the starch, and being then diluted with a pound of decoction of rice, was taken in the course of the day, in four doses. The ioduret of starch was borne perfectly well, and the dose was increased to an ounce and a half, two ounces, (containing forty-eight grains of iodine,) and two ounces and a half. The fæces were of their regular colour, and the urine, when treated with nitric acid, gave a yellow tint to white paper, or a deep blue when the paper was previously covered with starch: hence it appeared that the ioduret of starch was wholly absorbed. The dose was increased to three, and then to four ounces, (containing ninety-six grains of iodine,) but without lessening the disease. The patient now became disgusted with the remedy, and it was discontinued. In forty-eight days he took one hundred and thirty-nine ounces, or nearly nine pounds of ioduret of starch, containing 3336 grains, or nearly six ounces of iodine; being nearly sixty-six grains a day of this active substance.—*Id.*, *Ibid.*

